# Effects of Integrated Violence Intervention on Alexithymia, Cognitive, and Neurocognitive Features of Violence in Schizophrenia: A Randomized Controlled Trial

**DOI:** 10.3390/brainsci11070837

**Published:** 2021-06-24

**Authors:** Mei-Chi Hsu, Wen-Chen Ouyang

**Affiliations:** 1 Department of Nursing, I-Shou University, Kaohsiung City 82445, Taiwan; hsu6889@gmail.com; 2 Department of Geriatric Psychiatry, Jianan Psychiatric Center, Ministry of Health and Welfare, Tainan City 71742, Taiwan; 3 Department of Nursing, Shu-Zen Junior College of Medicine and Management, Kaohsiung City 82144, Taiwan; 4 Department of Psychiatry, College of Medicine, Kaohsiung Medical University, Kaohsiung City 80708, Taiwan

**Keywords:** repetitive violence, alexithymia, behavior, neurocognition, emotion regulation, schizophrenia, randomized controlled trial

## Abstract

Patients with schizophrenia and repetitive violence express core impairments that encompass multiple domains. To date, there have been few interventions integrating neurocognition, social cognition, alexithymia, and emotion regulation together as an approach to manage repetitive violence. The aim of this open-label randomized controlled trial was to examine more comprehensively the effectiveness of a novel Integrated Cognitive Based Violence Intervention Program on management of repetitive violence in patients with schizophrenia (vSZ). Sixty recruited patients were aged ≥20 years, diagnosed with schizophrenia for >2 years, had repetitive violent behavior within one year, and were psychiatrically hospitalized. The vSZ patients were randomly allocated to two groups and received either the intervention or treatment as usual. The intervention module, consisting of all defined 11 cognitive and social cognitive domains as well as emotion regulation, which were grouped into four modules. The intervention placed emphasis on the patients’ behavioral problems or intrinsic conflicts in relation to repetitive violence. The results indicate a statistically significant trend toward reducing impulsivity, anger with resentment, physical aggression, suspicion, and hostility (*p* < 0.05). The intervention significantly alleviated the intensity of cognitive failure, improved the management of alexithymic features and attribution styles and errors, and fostered adequate decision-making styles and emotion regulation capacity (*p* < 0.05). The intervention, when applied in conjunction with psychiatric standard care, could exert synergistic effects on alexithymia and cognitive, clinical, and neurocognitive features of repetitive violence in schizophrenia. This intervention provided patients a more active role to manage their violent behavior with the involvement of alexithymia.

## 1. Introduction

Schizophrenia is a debilitating mental disorder. During periods of active psychosis, there is a significant increase in risk of violence by as high as 7-fold or 2- to 8-fold for both male and female patients in comparison with the general public [1,2]. The definition of violence and aggression developed by the World Health Organization [3] is deliberately broad, to represent and describe violence very accurately in all forms with social and cultural diversity. WHO’s definition is also deemed to be one of the stronger existing definitions of violence. The present study adopted the WHO’s definition of violence and aggression: “The intentional use of physical force or power, threatened or actual, against oneself, another person, or against a group or community, that either results in or has a high likelihood of resulting in injury, death, psychological harm, maldevelopment or deprivation”. In this definition, aggression is subsumed under the definition of violence. Types of aggression recorded included verbal aggression, aggression toward property, self-harm/autoaggression, and physical aggression [4,5]. The American Psychiatric Nurses Association, Council for Safe Environments provides the clear definitions of these four types of violence [6]. Physical aggression is behavior intended to cause pain, physical harm, or death toward others. Verbal aggression includes verbal hostility, statements, or invectives to cause psychological harm to another through humiliation, intimidation, or devaluation. Aggression against property is broadly defined as a deliberate behavior of damage or destruction of property, or possessions of others. Autoaggression includes physical injury toward oneself, self-mutilation, or attempted suicide. The nature of violence, whether verbal or physical, is a major concern and therapeutic challenge for medical staff in the care of people with schizophrenia [7,8]. 

Some patients with schizophrenia commit violence in a repetitive manner [9]. Repetitive violence is often a complex and multifactorial construct that involves biological, psychosocial, situational and contextual factors and their combined effects [9,10]. Some studies used recurrent violent behavior as an interchangeable term for repetitive violence [10,11]. Furthermore, Herrera-Ferrá et al. [10] define repetitive violence (recurrent violent behavior) as a maladaptive, repetitive, and persistent pattern of violent behavior that inflicts injury, harm, death, and/or probable predisposition to subsequent criminality. Repetitive violence in patients with schizophrenia (vSZ) is generally associated with coexisting cognitive impairment, deficits in neurocognition (such as memory, motor function, and motor lapses in daily life, and perceptual-motor processing speed), impaired social cognitive function, and ineffective communication [12,13]. Neuro- and social cognitive deficits in schizophrenia are not only an enduring feature of the illness, but also a predictor of clinical and functional outcomes [14].

Core impairments underlying schizophrenia include multiple domains. More than three-quarters of patients with schizophrenia have some signs of cognitive impairment [15]. Rispaud et al. [16] have shown improvement in specific cognitive skills and functional ability in patients with schizophrenia during one year of cognitive rehabilitation. They demonstrated that improved processing speed skills and working memory could predict improvement in functional ability. Soyka [17] has suggested the association between neurocognitive deficit and risk of violence among people with schizophrenia. The degree of neurocognitive impairment can also affect processing speed, which can be a predictor of successful competency restoration. Thus, improvement in message delivery, increased speed, and information processing efficiency can prevent the deterioration of brain neurocognitive function and improve cognitive performance. 

Like neurocognitive deficits, social cognitive deficits, including impairments in many domains such as emotional processing, and attributional bias, are prevalent among people with schizophrenia [12,18]. There are studies that linked social cognition deficits with schizophrenia-related violence/aggression [18,19]. For example, inadequate attributions increase the risk of violence [8]. Both neuro- and social cognitive impairments have great impacts on functional outcomes of daily life in both early [20,21] and chronic phases [18,22]. 

To date, neuro- and social cognitive approaches in managing violence in people with schizophrenia remain relatively unexplored. There are studies that have evaluated the relationship between changes in cognition and functioning for development of suitable cognitive and psychosocial interventions for schizophrenia [23,24,25]. Others have examined the effectiveness of different intervention programs designed for these clinical problems. However, results from these studies were inconsistent and sometimes paradoxical. The disparity in findings may be due to diagnostic heterogeneity, use of performance-based outcome measures of function, the nature of the cognitive treatments selected, stage of illness, and standardization in quantification of violence scale. The inconsistent effectiveness of interventions could also be due to differences in training regimens, measurement indices, and behavioral changes. For example, there are integrated neurocognitive therapy [26,27], cognitive remediation therapy [28], social cognitive training [29,30], combined computerized social cognitive training, neuroplasticity-based auditory training [31], and integrated psychological therapy [32,33]. 

Emotion dysregulation is also associated with violent behavior [34]. Velotti et al. [35] have shown that emotion dysregulation influences the link between alexithymia and aggression. Negative emotionality in people with schizophrenia reflects a lack of emotion regulation skills. Individuals deployed several regulatory strategies or relied on cognitive processes to regulate emotion [18]. 

Violent behavior could also be caused by personality traits, such as alexithymia, and specific clinical psychotic features [36,37]. Alexithymia is not classified as a mental disorder/personality disorder [38]. Alexithymia, which refers to a lack of the ability to process emotion in response to stress and other extreme events, is a risk factor that predates violence in schizophrenia [18,39]. Velotti et al. [35] evaluated in psychiatric patients the role of personality traits in aggression, and suggested that the specific role of different components of alexithymia should be considered in developing future aggression regulation programs. Alexithymia is also related to social cognition deficits, emotion regulation, communicative deficit, and externalizing behavior (such as increased expressions of anger, poor impulse control, heightened negative emotionality, and higher trait aggression). Under distress, individuals with alexithymia and low capacities to regulate emotions can easily feel threatened and therefore react violently. 

Over the past decades, evidence of the association between schizophrenia and violence has accumulated (e.g., [40]), thereby identifying a multitude of relevant risk factors. Risk factors of violence mainly concern individual diagnoses such as schizophrenia, genetic influences between mental illness and violence, a history of victimization, comorbid substance abuse, alcohol use, antisocial behavior, poor adherence to treatment, and overall symptom severity, etc. [36,41,42,43]. Comorbidity with substance abuse is the most important determinant of aggressive behaviors among persons with schizophrenia [36,44,45]. Other, more disorder-specific determinants, such as persecutory delusions and hallucinations in schizophrenia, are also of importance to violent behavior [36]. Repetitive violent acts are also associated with the coexistence of cognitive impairment, hostility, patient history or experiences of violence, or disorganization with impaired reality testing [9,36]. 

Cultural, racial, and ethnic contexts are also critical and highly influential in analyzing violence [41,46]. Studies have drawn attention to the role of ethno-racial trauma in the experiences of immigrants and racial minorities [46]. Indeed, acts of violence may vary greatly across cultural contexts. However, bullying or aggression that target cultural, racial, and ethnic differences lead to physical health conditions and emotional and mental and psychological distress [46].

Currently, studies focused on how alexithymia influences the control of emotional arousal in patients with schizophrenia are scarce. It is known that deficits in neuro- or social cognitive functions bring about poor impulse control, problem solving, decision-making, and ability to manage stress [47]. Thus, there is a great need to better understand the complex dynamics among alexithymia, neuro- and social cognitive functions, and violent behavior. However, very few studies have systematically examined whether an integrated violence intervention might have a beneficial influence on violent behavior by improving emotion regulation and the neuro- and social cognitive functions. These variables are reasonably intertwined. Thus, improvement of both neurocognitive functions, such as perceptual-motor processing speed, and social cognitive functions, and also effective emotion regulation strategies, should be an even more relevant approach for management of repetitive violence.

A randomized controlled trial was carried out to evaluate in a more comprehensive way the effects of the novel Integrated Cognitive Based Violence Intervention Program (ICB-VIP) on violence management, and the mechanisms and interrelationships among alexithymia, neurocognitive and social cognitive functions, emotion regulation, and violence in vSZ patients. The research hypothesis was that ICB-VIP, when applied in conjunction with psychiatric standard care, could exert synergistic effects on alexithymia, cognitive, clinical and neurocognitive features of repetitive violence in schizophrenia.

## 2. Materials and Methods

### 2.1. Study Design

The present study was an open-label randomized controlled trial carried out at a psychiatric center in Taiwan from 2018 to 2019. We collected data at 3 different time intervals: prior to intervention (T1), after intervention (T2), and 1-month follow-up after intervention (T3).

### 2.2. Participants and Recruitment

Patients were selected on the basis of the following inclusion criteria: 20 years or older, a confirmed psychiatric diagnosis of schizophrenia (F20.0, F20.1, F20.3, F20.5, F20.89, and F20.9) using the ICD-10 coding system for more than 2 years by psychiatrists, psychiatrically hospitalized, record of repetitive violent behavior within 1 year, and voluntary submission of written informed consent. Patients were excluded from the study if they had a confirmed psychiatric diagnosis of catatonic schizophrenia (F20.2) and schizophreniform disorder (F20.81) due to poor diagnostic stability, were aged ≥65 years, or had uncorrected visual or hearing impairments, neurological disorders other than schizophrenia, developmental disability, signs of intellectual disability, personality disorders, a record of current substance abuse (except for caffeine), or a history of substance abuse (except for alcohol, nicotine, or caffeine). A comprehensive health assessment and clinical evaluation of substance abuse were conducted upon study enrollment. In Taiwan, the legal age of adulthood in the Civil Code was 20 at the time of our data collection. Thus, patients aged between 18 and 20 years were not recruited. Furthermore, elderly patients could have signs of cognitive and neuropsychological impairment, behavioral and psychological symptoms of dementia (BPSD), and/or poor physical functioning comorbid with undetected delirium. These health conditions may be considered as potential confounding factors in the present study. Adult patients were targeted in this study because violence is elevated in older adolescents and adults with schizophrenia [48]. Their violence may cause harm or injury to their family and/or other people.

The research team consisted of a psychiatrist, allied health professionals, and nurses and nursing educators with specialized skills and expertise working together in a committed attitude to ensure good quality of the study. The research team members cared for and interacted attentively with patients.

### 2.3. Sample Size and Estimated Study Power

Based on a desired power of 80%, α = 0.05, and effect size = 0.62, an appropriate sample size of 60 subjects (30 participants each in experimental and control groups) was selected for the current study.

### 2.4. Randomization

After their written and signed informed consent was obtained, all inpatients were randomly allocated to either the ICB-VIP or Treatment as Usual (TAU) groups. TAU is referred to as a standard of care given to patients that involves a broad range of mental health treatments in clinical practice (e.g., medication, regular ward group and/or individual therapy, case management). The randomized allocation schedule was generated by a computer, and treatment assignments were sealed in sequentially numbered opaque envelopes by a trained nurse. The researchers opened the envelopes and then allocated the patients to their assigned groups. 

### 2.5. Intervention

The ICB-VIP modules consisted of all 11 defined cognitive and social cognitive domains as well as emotion regulation, which were grouped into 4 modules. The elements and strategy of the intervention capitalized on, and were influenced by, several cognitive rehabilitation programs, such as the Neuropsychological Educational Approach to Remediation (NEAR) [49], Compensatory Cognitive Training manual [50], and relevant strategies [51,52]. 

ICB-VIP is an individualized and specially created program. It emphasizes neuro- and social cognitive functions through repeated training based on vSZ patients’ cognitive profiles predominantly, reasoning and problem solving, cognitive biases, violence attribution, and emotion regulation. The structure applied to each module is identical, starting with an introduction session, followed by a compensation session, and ended with a discussion/restitution session. Each session lasted approximately 1–1.5 h.

ICB-VIP closely linked each patient’s violent behavioral problems. Prior to each session, all patients were requested to participate in a 10-min bridging interview session. To meet the special needs of individual vSZ patients, all sessions were presented at different levels of complexity and difficulty. The design of individualized sessions according to each patient’s cognitive functions and violent behavioral problems makes ICB-VIP a suitable intervention for vSZ patients. 

The core contents of the intervention centered first on the development of individual strategies to improve neuropsychiatric capacity (such as speed of processing, cognitive control during provocative situations), identification of basic emotions, the use of environmental manipulation, and cognitive flexibility. Secondly, the intervention helped patients to implement problem solving, distinguish between facts and assumptions, identify how their characteristics in interacting with other people produce violent events, and develop skills training to manage violence, communication, and impulse control and achieve other rehabilitative goals such as problem-solving, the development of conflict resolution strategies, and recognition and management of violence. These helped patients reflect on the occurrence of misunderstandings during violent situations and understand how they themselves handle such situations.

A drill-and-practice approach such as in vivo exercises, which serve to promote the transfer of learned cognitive skills and emotional management skills into patients’ daily activities, was carried out by practicing role-playing strategies in real life. As the participant improved, the exercises also increased progressively and systematically in difficulty. 

### 2.6. Outcome Measures

This study incorporated a comprehensive battery of assessments to measure the effect of intervention on neuro- and social cognition, severity of alexithymia, decision-making style, emotion regulation, clinical symptoms, and domains of violence. Selection of outcome measures was in accordance with the basis for designing the intervention. 

#### 2.6.1. Measures of Perceptual Motor Processing Speed

1. Finding As task: Each Chinese character comprises a radical and other parts; for example, Chinese characters such as 叩 and 合 contain a “口” radical. A pile of 105 Chinese characters was included in the test. Patients had to pick from the pile the characters containing a “口” radical. Total time required to complete this task was recorded [53].
2. Number comparison: Participants identified identical or mismatched number pairs (3 numbers) [54]. Response time required to match each pair was recorded.
3. Copying task: Fifty Chinese characters and 50 digits were shown on the test sheet. The patient was asked to copy the characters and numbers that were presented on paper as fast and accurately as possible. The score for this task was the average time required per character and number [55]. The test used a stopwatch (second or centisecond, cs) to record the completion time for each stage.

#### 2.6.2. Cognitive Failure Questionnaire (CFQ) 

Cognitive failures were assessed using a cognitive failure questionnaire (CFQ) [56]. The CFQ is a 25-item questionnaire that measures the frequency of daily cognitive failures and errors or lapses in everyday tasks in the past 6 months (Cronbach’s α = 0.95). Each item was scored from 0 (“never”) to 4 (“very often”). Higher total CFQ scores indicated more subjectively experienced cognitive failures. 

#### 2.6.3. Reasoning and Thinking Styles (Rationale–Experiential Inventory, REI) 

REI was used to measure an individual’s rational and experiential thinking styles [57]. There are 20 questions for each style, which can be further divided into 10-item subscales to assess an individual’s ability and engagement in the given style. Each item is rated on a 5-point Likert scale, ranging from 1 (definitely not true of myself) to 5 (definitely true of myself). The total scores of rational and experiential thinking styles were summed up with a higher score indicating a greater tendency to the style measured. In the present study, the internal consistencies for the REI scale and its subscales were high with all Cronbach’s α ≥ 0.87. 

#### 2.6.4. Alexithymia (Toronto Alexithymia Scale-20, TAS-20) 

TAS-20, a self-reporting questionnaire, was used to measure levels of individual alexithymia [58]. The questionnaire includes 20 items covering 3 dimensions: difficulty in identifying feelings (DIF); difficulty in describing feelings (DDF); and externally orientated thinking (EOT). Each item was rated on a 5-point Likert scale, with a total score ranging between 20 and 100. The distinctive cutoff scores to indicate the degree of alexithymic problems were as follows: ≤50 indicated no alexithymia, 51–60 indicated borderline alexithymia, and ≥61 indicated alexithymia [58]. 

#### 2.6.5. Violence Attributions (Attribution Questionnaire, AQ) 

The AQ is a 6-item self-reporting assessment that measures 2 dimensions of violence attributions: internal/external attributions of responsibility/blame for the violence, and attribution of causality of violence [59]. The measure was rated on a continuum scale of 1 (e.g., not at all likely; not at all able, etc.) to 7 (e.g., totally due to me; totally likely; totally able, etc.). 

#### 2.6.6. Aggression Frequency (Modified Overt Aggression Scale, MOAS) 

The MOAS was used to evaluate the severity and frequency of violent behavior in 4 categories of violence: verbal violence, physical violence, aggression against property, and autoaggression [5]. Each category consisted of 5 items.

#### 2.6.7. Violence/Aggression (Buss-Perry Aggression Questionnaire, AGQ) 

The 29-item aggression questionnaire was used to measure aggression in patients [60]. It was divided into 4 categories: physical aggression, verbal aggression, anger, and hostility. Each participant ranked all items on a continuum scale from 1 (extremely uncharacteristic of me) to 5 (extremely characteristic of me). The sum of scores ranged between 29 and 145, with higher scores indicating higher levels of aggression. 

#### 2.6.8. Severity of Clinical Symptoms (Positive and Negative Syndrome Scale, PANSS) 

The PANSS was used for measuring negative and positive symptom severity of patients [61]. Severity ratings of clinical symptoms were compiled from the structured interview with patients, information provided by health professionals of the hospital treatment team, and chart reviews. Each PANSS item was rated on a 7-point scale: 1 = absent, 2 = minimal, 3 = mild, 4 = moderate, 5 = moderately severe, 6 = severe, and 7 = extreme. The absence of symptoms was rated as 1. Lower scores indicated better clinical status.

### 2.7. Intervention Fidelity, Validity, and Reliability

We assessed the fidelity, reliability, and validity of the intervention following the procedure reported by Borrelli [62]. The components of fidelity focused primarily on 3 dimensions: researcher training and development, mode of delivery of intervention, and the ways participants were recruited and assigned to the intervention. 

### 2.8. Ethical Considerations 

The protocol of this intervention trial was approved by the Institutional Review Board (IRB) of Jianan Psychiatric Center, Ministry of Health and Welfare, Taiwan (registered No.17-030). A signed written informed consent from each participant was secured prior to treatment. As patients were relatively vulnerable, a thorough psychiatric evaluation of potential participants was performed by the research team before the study began. Throughout the study period, all researchers ensured the rights, health, and well-being of patients following the ethical principles for medical research on human beings laid down in the Declaration of Helsinki. 

### 2.9. Data Analysis

Statistical analyses were performed using the software SPSS version 22.0, for Windows (IBM). Normality of raw data was checked, and outliers removed. Descriptive statistics, expressed as mean and standard deviation (SD), were used for continuous demographic variables; at the same time, frequency was used for categorical demographic variables. To test homogeneity between groups regarding their distribution in categorical demographic data, the chi-square test (χ^2^) was applied. To examine whether between-group differences in all outcome measures were significant or not, the independent *t*-test was used. To determine whether interventions affected within-between groups and group-by-time interaction, a repeated-measures analysis of variance (ANOVA) was included in the outcome measures. The significance level was set at *p* < 0.05. 

## 3. Results 

### 3.1. Basal Characteristics of the Participants

A total of 63 vSZ patients were screened and recruited. Three patients refused to participate in the study. The reasons were that they were not interested. A total of 60 patients (95.2%) completed the study. The mean ages (±SD) of patients in the ICB-VIP and TAU groups were 44.57 (±4.66) and 44.27 (±4.27), respectively. Age distributions were similar between the two groups (*t* = 0.593, *p* = 0.796). None of the participants consumed betel nut or any substance other than caffeine, alcohol, or nicotine before admission. All other demographic variables were also similar between the ICB-VIP and TAU groups (*p* > 0.05) (Table 1). The MOAS scores, used to assess the severity and frequency of violent behavior, were high in all vSZ. There were no differences between the ICB-VIP (10.20 ± 7.5) and the TAU groups (9.43 ± 6.63) (*t* = 0.416, *p* = 0.679). The committed episodes of violence in both groups were also comparable (*p* > 0.05). 

### 3.2. Effects of Intervention on Violence and Violence Attribution

Table 2 shows that the aggression scores in the ICB-VIP group, compared to those in the TAU group, were significantly reduced at T2 and T3 (*p* < 0.05). A statistical trend in reducing anger with resentment, physical aggression, suspicion, and hostility was also observed in the ICB-VIP group (*p* < 0.05) compared to the TAU group. However, ICB-VIP intervention had no significant effect on verbal aggression (*p* > 0.05) at T2.

Regarding violence attribution, no significant differences were found between the two groups before the intervention (*p* > 0.05). However, the violence attribution scores in the ICB-VIP group changed significantly from T2 and thereafter (*p* < 0.05). 

### 3.3. Effects of Intervention on Severity of Clinical Outcomes

The intervention also demonstrated significant reductions in positive symptoms (*t* = −4.547, *p* = 0.000) and general psychopathology symptoms (*t* = −3.743, *p* = 0.000). This was reflected by a significant decrease in overall global symptom score at follow-up (*t* = −4.968, *p* = 0.000). Additionally, significant reductions of the blunted affect (item 1), social withdrawal (item 4), and lack of spontaneity (item 6) were found in the ICB-VIP as compared to TAU at follow-up. However, ICB-VIP did not improve the negative symptoms (*p* > 0.05). 

### 3.4. Effects of Intervention on Neurocognition, Reasoning Style, and Decision-Making

Results in Table 3 show a significant improvement in cognitive failures at T2 (*t* = −3.550, *p* = 0.001) and T3 (*t* = −4.466, *p* = 0.000) in the ICB-VIP compared to TAU group. The intervention also significantly improved rational thinking style at T2 (*t* = 8.352, *p* = 0.000) and T3 (*t* = 11.657, *p* = 0.000). Scores of rational ability and rational engagement were significantly improved in the ICB-VIP group as compared to the TAU group at T2 and T3 (*p* = 0.000). Statistical analysis also showed reducing experiential ability and experiential engagement in the ICB-VIP group at T3 (*p* < 0.01) but not at T2 (*p* > 0.05). The overall experiential thinking style scores were decreased only at follow-up (*t* = −4.411, *p* = 0.000).

### 3.5. Effects of Intervention on Alexithymia

Results in Table 4 show that ICB-VIP as compared to TAU had significant effects on global alexithymia at both T2 (*t* = −3.462, *p* = 0.001) and T3 (*t* = −4.261, *p* = 0.000). When different domains of alexithymia were assessed, the ICB-VIP group significantly improved DIF compared to the TAU group at T2 and T3 (*p* < 0.05). The EOT scores were progressively decreasing in the ICB-VIP group, but were increased in the TAU group at T2. Consequently, the differences became significant at T3 (*p* < 0.05). The ICB-VIP group also improved its DDF scores at T2 (*t* = −2.202, *p* < 0.05), but this effect was not sustained at T3 (*p* > 0.05).

### 3.6. Effects of Intervention on Emotion Regulation Outcomes

In terms of emotion regulation, scores of the reappraisal subscales were similar between groups at T2 (*p* > 0.05), but were significantly higher at T3 (*t* = 6.904, *p* < 0.05) in the ICB-VIP group compared to the TAU group. Results also showed improvement in the scores of the suppression domain at T2 (*t* = 2.751, *p* = 0.008) and T3 (*t* = 4.487, *p* = 0.0005). The scores decreased progressively in the ICB-VIP group but increased in the TAU group at T3. 

### 3.7. Repeated-Measures Effects of Intervention on Study Outcomes

A two-way repeated measure ANOVA was used to compare the mean differences between the ICB-VIP and TAU groups in order to determine whether any change in aggression and study outcomes resulted from interaction between intervention and time. Results in Table 5 show that the time factor significantly affected intervention outcomes. At all three test time intervals, aggression, outcome of severity of clinical symptoms, neurocognition, reasoning style and decision-making, and social cognition and emotion regulation were significantly different between these two groups (*p* < 0.000). As time increased, these differences became greater. Significant improvements over time in aggression (F = 12.311, *p* = 0.000) and the study outcomes (*p* = 0.000) were also observed in the ICB-VIP group compared to the TAU group. 

## 4. Discussion

As far as we are aware, our work is one of only a handful of studies that have investigated how changes in multiple domains that were related to one another were put in the context of this novel intervention for management of repetitive violence. It is known that when investigations across multiple domains, such as neuro- and social cognition, personality traits, attributional style, decision-making style and preferences, and severity of clinical symptoms are involved concurrently, the intervention becomes complex. We analyzed all of the multi-tasking assessments, which were not usually performed by other studies. These findings are also in agreement with previous studies showing that combined treatment of neuro- and social-cognitive clinical intervention could produce larger effects on functional outcome [12,32,33] and violence management. 

As hypothesized, we found that compared to TAU, ICB-VIP showed significant improvements in some of the neuro- and social cognitive domains, symptoms, and functional outcomes. Particularly, the positive effects of ICB-VIP on these neurocognitive domains and emotion perception were consistent with findings from other studies [12,30,32]. Assessment of various factors at three different time points precisely provided us the opportunity to evaluate the longitudinal change of the intervention effects. Although there were no significant effects on perceptual-motor processing speed, we found additional significant improvements in cognitive failure at follow-up. There are several explanations of possible treatment-related improvements in repetitive violence and outcome measures. First, ICB-VIP augments progress in producing faster cognitive gains and greater functional gains. Improvement in neurocognition plays a role in reducing violence [63]. Second, the combination of neuro- and social cognition as well as emotion regulation can be a novel and viable approach to manage violence in schizophrenia. Third, gains from ICB-VIP in some outcomes appear to be sustainable throughout the follow-up period.

Neurocognitive impairments are known to be associated with violence among inpatients with schizophrenia [47,64]. An intervention that improves neurocognition might lead to reduced violence. Indeed, we found in this study that reductions in violence correlated well with improved neurocognitive scores. Cognitive failure also makes a person more vulnerable to stress. Thus, improvements in areas of cognitive failure, such as attentiveness and memory in everyday life, can reduce vulnerability to stress. Furthermore, cognitive alterations in schizophrenia may have an impact on attention and memory. Our study showed that implementation of ICB-VIP and related strategies could improve attention and immediate logical memory, which increase the ability of patients to learn, maintain, and recall during stressful situations. 

Improvements in specific social cognitive functions such as alexithymia features, attributional style, decision-making style, and preferences can also be related to reductions in violent actions. Alexithymia is one of the factors that precedes violence [65,66]. As shown in this study, vSZ patients had significantly higher alexithymia scores at baseline. DIF of alexithymia is the main factor related to violence. Similarly, alexithymia is associated with aggressive behavior because of emotional avoidance and emotional inexpressivity [39,67,68], which is negatively correlated with the ability to cope with stress [69]. Although alexithymia is not a personality disorder, alexithymia may be associated with personality dysfunction [70]. Personality disorder is one of the common risk factors for violence [71,72,73]. Studies have provided evidence of personality disorders [36] such as antisocial personality disorder [74] and borderline personality disorder [37] representing a significant risk for violence.

Our intervention was demonstrated to be able to effectively ameliorate emotional processing in vSZ patients. Adaptive emotion regulation requires skills such as emotional awareness and acceptance and control of impulsive behaviors. People who have poor ability to understand their feelings and difficulty in controlling their violent tendencies with conscious effort tend to act impulsively [75,76]. As a result, poor communication often occurs. We showed in this study that the ICB-VIP could guide vSZ patients in learning how to be more attentive to personal feeling states and how to identify emotions in others. 

Differences in violent behavior may relate to individual differences in attributional styles. Schizophrenia patients often exhibit hostile attributional biases; they tend to perceive others’ intentions as hostile and the cause of violence. Development of these inadequate attribution styles or errors increases later violent behavior [77,78]. How to alter the victim-blaming attributions is a challenge for healthcare professionals who work in violence intervention programs [77]. Our findings show that ICB-VIP was effective in changing vSZ patients’ attributions of violence. After intervention, patients reduced their victim-blaming attributions and admitted being responsible for violence, which reflected the effectiveness of the intervention [7,78]. 

One of the most important impacts of violence depends on an individual’s decision-making style and preferences and actions to reduce violence [8]. To effectively control violence, neurocognitive functions such as ability to generate alternative responses to stressful situations and select appropriate responses to stress are essential. Thus, vSZ patients need an integrated intervention that offers suitable strategies to confront the complexities of their own violent behaviors. Our study found improvements in facilitating thinking and decision-making after intervention. Improving cognitive capacity enhances decision-making and action selection. One possibility is that improvements in neuro- and social cognition and emotion regulation represent a necessary prerequisite for violence management. Improvements increase the ability of vSZ patients to reappraise and select better responses during stressful situations. 

The effect of the ICB-VIP on violence reduction may also relate to improvements in emotion regulation. Improvements in emotion regulation can be accomplished through adopting different emotion regulation strategies [79]. For example, studies have shown that one who uses deliberate emotion regulation strategies can effectively maintain better emotion experience and proper behaviors when facing difficult emotions [80]. Schizophrenia patients have difficulty identifying emotions, specifically, negative emotions [81,82]. Our results support previous findings [83,84] that emotion regulation could be improved by the appropriate intervention, even for patients with a history of violence. One explanation of the effectiveness is that vSZ patients learned de-escalation techniques for the management of violence and aggression through ICB-VIP. In the present study, to reduce their aggressive impulses, vSZ patients after intervention were able to use emotion regulation strategies such as trying to down-regulate their negative emotions, balancing anger during interpersonal conflicts with family members and other persons, or shifting attention from a distressing situation. These techniques enhanced the learning capacity necessary to improve their anger management, regulate their emotions, and express displeasure appropriately. 

Studies have also suggested that vSZ patients, compared to non-vSZ, are more profoundly impaired in executive functions and working memory [17,85]. These impairments may limit their learning ability to benefit from competency restoration and psychiatric rehabilitation programs [86]. 

With regard to symptoms, the ICB-VIP significantly improved the PANSS outcomes at follow-up. However, the benefits appeared only in the negative and excitement/conceptual disorganization domains. As shown by Candini et al. [87], violent patients have lower levels of negative symptoms compared to controls. Improvements in negative symptoms shown in our study were also consistent with those reported in other studies [88,89]. In this study, the ICB-VIP and not the TAU group showed improvement in hostility scores. Hostility is one of risk factors for repetitive violence or other behavioral disturbances [8]. Generally, vSZ patients with hostility have difficulty in dealing with people under stressful circumstances, and they are likely to exercise violence towards family members and other people whether or not they are angry.

In this study, the ICB-VIP was highly accepted by patients. This could be attributed to the fact that the ICB-VIP places strong emphasis on the enhanced management of intrinsic problems or conflict, and takes seriously the patients’ daily-living experiences. The findings provide prompt and important evidence that neuro- and social cognitive deficits may serve as viable treatment targets in our efforts to reduce emotion regulation abnormalities and subsequently, repetitive violence in schizophrenia. The distinction between the ICB-VIP and cognitive therapy is that the ICB-VIP integrates neurocognition, social cognition, alexithymia, and emotion regulation together as an important approach for managing repetitive violence. Health professionals would need to undergo training in integrated violence management. The effective management of repetitive violence can be an important part of clinical practice that complements already existing concepts, values, knowledge, and skills for patients. Another value of this intervention is that vSZ patients were provided a more active role in controlling their violent behavior and aggressive feelings by improving alexithymia.

Patients with intellectual disability (ID) may have significant signs of cognitive, learning, and communication problems, difficulty remembering things, or difficulty with problem-solving, etc. These conditions may be considered as potential confounding factors or may possibly influence the completion of study tasks. Furthermore, studies have found that individuals with intellectual disabilities are at particularly high risk of violence compared to the general population [90,91,92]. They had a 3-fold increase in risk of violent victimization, and a 10-fold increase in risk for sexual victimization [92]. Experiences of violent victimization among adult individuals with both ID and comorbid mental illness may be associated with their cognitive impairment, communication difficulties, higher levels of dependence, and discrimination [90,91,92]. Thus, because of the role of intelligence in the violent behavior of psychotic patients, they were not included in the present study. 

Some limitations of this study must be considered. First, the present study did not collect data regarding some individual clinical information, such as year of onset of schizophrenia, medication adherence, and the dosage of antipsychotic regimen for statistical analysis. Dose profiles for mean changes from baseline were also not evaluated. The lack of individual dosimetry data for confounding factors remain a limitation in the present study. Thus, the analyses may not have accounted fully for how the clinical effects could be affected by drugs between those in the experimental and control groups. Second, a three-arm design with an active control group and a usual-care control group is recommended because it can assist in controlling for the nonspecific features of an intervention. Third, the follow-up period (one month) was relatively short. Longer follow-up periods (>6 months) are recommended in future investigations of this kind, so that the complex neuro- and social cognitive functions can be further improved and the treatment mechanisms, neurocognitive and behavioral changes, and effectiveness better confirmed.

Based on the research findings and limitations of this study, some suggestions for future study and research are as follows. First, future studies should include the outcome measures such as personal and social performance (PSP) and/or global assessment of functioning (GAF) for a better assessment of patients regarding their autonomy in activities of daily living (ADL) and psychosocial functioning. Second, there is a need to investigate quality of life (QOL) among this population in future studies. Third, the implementation of a comprehensive, diagnostic screening approach that includes broad laboratory testing such as cranial magnetic resonance imaging and electroencephalography investigations is worthwhile and recommended in order to detect and exclude secondary, organic psychoses [93]. Larger studies are warranted. Fourth, it is also recommended to use the psychopathy checklist—revised (PCL-R) or even the historical, clinical and risk management −20 in predicting violent recidivism among persons with schizophrenia. Fifth, testosterone has been used as a biological marker for aggression and violence. We did not have data on testosterone levels in our participants. In building upon pre-existing neuropsychiatric models of violence, further studies are recommended to investigate the role of plasma levels of testosterone in violent behavior.

## 5. Conclusions

The present study highlighted the importance of having neurocognitive and social cognitive functions and emotional recognition capacity considered together for enhancing the effect of clinical intervention in the management of repetitive violence in vSZ patients. Our study demonstrated that this integrated violence intervention could alleviate the intensity of cognitive failure, improve specific neuro- and social cognitive functions, manage alexithymia features, attribution styles, and errors, and foster adequate decision-making styles and preferences, as well as emotion regulation capacity. Thus, the present study provided information on how these functions and symptoms change over time and whether these changes co-occurred. This intervention empowers patients to recognize and acknowledge different aspects of repetitive violence in a more nuanced manner, inspire more patient self-management, and thus, result in a greater movement toward an effective approach to violence prevention.

## Figures and Tables

**Table 1:** Characteristics of patients at recruitment.

		ICB-VIP		TAU		χ^2^	*p*
		*n*	%	*n*	%		
Gender	Male	16	53.3	17	56.7	0.067	0.795
	Female	14	46.7	13	43.3		
Education	Primary school/below	1	3.3	3	10.0	1.667	0.435
	Secondary/high school	25	83.3	25	83.3		
	Junior college/college	4	13.3	2	6.7		
Marriage status	Single	25	83.3	27	90.0	2.007	0.354
	Married	3	10.0	3	10.0		
	Separated/divorced	2	6.7	-	-		
Occupation	Unemployed	24	80.0	23	76.7	0.132	0.936
	Employed	6	20.0	5	23.3		
Religion	None	14	46.7	15	50	0.388	0.824
	Buddhism/Taoism	14	46.7	12	40		
	Christian/others	2	6.7	3	10		
Family monthly income (NT$)	<25,000	17	56.7	22	73.3	2.170	0.338
	>25,001~50,000	13	43.3	8	26.7		
Smoking	Yes	11	36.7	12	40.0	0.071	0.791
	No	19	63.3	18	60.0		
Level of smoking	1–5 cig/day	5	45.5	4	33.3		
	6–10 cig/day	5	45.5	6	50.0		
	11–15 cig/day	1	9.0	2	16.7		
	16–20 cig/day	-	-	-	-		
	21–30 cig/day	-	-	-	-		
	>31 cig/day	-	-	-	-		
Alcohol use	Yes	4	13.3	5	16.7	0.131	0.718
	No	26	86.7	25	83.3		
Drinking frequency	≤1 time/month	-	-	2	40.0		
	2–4 times/month	4	100	3	60.0		
	1–3 times/week	-	-	-	-		
	≥4 times/week	-	-	-	-		
Violent behavior							
Verbal violence	Yes/no	25/5	83.3/16.7	28/2	93.3/6.7	1.456	0.228
Physical violence	Yes/no	17/13	56.7/43.3	13/17	43.3/5637	1.067	0.302
Aggression against property	Yes/no	14/16	46.7/53.3	12/18	40.0/60.0	0.271	0.602
Auto-aggression	Yes/no	4/26	13.3/86.7	6/24	20.0/80.0	0.480	0.488

Note: ICB-VIP: Integrated Cognitive Based Violence Intervention Program; TAU: treatment as usual. cig: cigarettes.

**Table 2:** Pre-post-intervention and follow-up data for violence and severity of clinical outcomes.

ICB-VIP (ICB) vs. TAU	Baseline (Time 1)				Post-Intervention (Time 2)				Follow-Up (Time 3)			
	ICB	TAU	*t*, *p* ^a^	95% CIs	ICB	TAU	*t*, *p*^a^	95% CIs	ICB	TAU	*t*, *p*^a^	95% CIs
Aggression												
Anger with resentment	22.43 ±3.97	22.40 ±3.78	0.033, 0.974	−1.971, 2.038	20.83 ±3.31	22.33 ±1.62	−2.227, 0.030	−2.848, −0.152	19.23 ±2.34	21.37 ±2.23	−3.607, 0.001	−3.317, −0.949
Verbal aggression	15.13 ±3.31	15.00 ±2.86	0.167, 0.868	−1.469, 1.736	14.40 ±3.82	15.50 ±3.00	−1.238, 0.221	−2.878, 0.678	14.53 ±2.22	15.83 ±2.60	−2.081, 0.042	−2.551, −0.049
Physical aggression	27.23 ±7.47	28.33 ±3.67	−0.723, 0.472	−4.144, 1.944	23.50 ±5.76	28.43 ±3.50	−4.007, 0.000	−7.398, −2.469	23.43 ±3.89	26.53 ±3.83	−3.109, 0.003	−5.096, −1.104
Suspicion, hostility	23.40 ±4.68	24.27 ±2.74	−0.875, 0.385	−2.849, 1.115	21.63 ±3.69	24.93 ±2.79	−3.906, 0.000	−4.991, −1.609	20.63 ±3.00	24.90 ±2.74	−5.746, 0.000	−5.753, −2.780
Total	88.20 ±16.31	90.00 ±10.18	−0.513, 0.610	−8.829, 5.229	79.60 ±13.62	90.70 ±8.42	−3.795, 0.000	−16.954, −5.246	77.83 ±8.91	88.63 ±5.24	−5.719, 0.000	−14.580, −7.020
Violence attribution	22.27 ±3.32	24.07 ±3.75	−1.965, 0.054	−3.633, 0.033	25.47 ±3.62	23.67 ±3.10	2.066, 0.043	0.056, 3.544	26.33 ±2.69	24.57 ±2.45	2.652, 0.010	0.433, 3.100
Clinical symptoms ^b^												
Positive	23.40 ±3.82	22.40 ±1.56	1.327, 0.190	−0.509, 2.509	19.53 ±4.84	21.10 ±3.99	−1.367, 0.177	−3.860, 0.727	14.90 ±3.67	19.13 ±3.54	−4.547, 0.000	−6.097, −2.370
Negative	21.13 ±7.06	22.10 ±7.86	−0.501, 0.618	−4.830, 2.896	17.63 ±4.70	20.47 ±7.08	−1.824, 0.073	−5.942, 0.275	16.43 ±4.83	18.23 ±5.30	−1.373, 0.175	−4.424, 0.824
General	40.57 ±7.89	41.07 ±9.18	−0.226, 0.822	−4.926, 3.926	33.37 ±6.41	38.27 ±7.26	−2.770, 0.008	−8.441, −1.359	28.73 ±5.44	35.37 ±8.03	−3.743, 0.000	−10.181, −3.086
Total	85.10 ±12.75	85.57 ±13.76	−0.136, 0.892	−7.326, 6.393	70.53 ±11.34	79.83 ±11.33	−3.176, 0.002	−15.161, −3.439	60.07 ±9.55	72.73 ±10.18	−4.968, 0.000	−17.770, −7.563

Note: Values are expressed as means ± standard deviations. ^a^: Examined by independent *t*-test between groups; ^b^: PANSS = positive and negative syndrome scale; ICB-VIP: Integrated Cognitive Based Violence Intervention Program; ICB-VIP is abbreviated as ICB in table; TAU: treatment as usual.

**Table 3:** Pre-post-intervention and follow-up data for neurocognition, reasoning style and decision-making.

ICB-VIP (ICB) vs. TAU	Baseline (Time 1)				Post-Intervention (Time 2)				Follow-Up (Time 3)			
	ICB	TAU	*t*, *p* ^a^	95% CIs	ICB	TAU	*t*, *p*^a^	95% CIs	ICB	TAU	*t*, *p*^a^	95% CIs
Cognitive failure ^b^	59.60 ±5.24	57.60 ±4.43	1.596, 0.116	−0.509, 4.509	51.67 ±5.26	56.97 ±6.26	−3.550, 0.001	−8.289, −2.311	46.53 ±6.52	54.43 ±7.16	−4.466, 0.000	−11.441, −4.359
Reasoning and decision-making ^c^												
Rational	5.94 ±0.42	6.09 ±0.31	−1.553, 0.126	−0.343, 0.043	6.84 ±0.42	6.00 ±0.34	8.352, 0.000	0.636, 1.037	7.57 ±0.50	6.18 ±0.41	11.657, 0.000	1.151, 1.629
Experiential	7.55 ±0.42	7.49 ±0.43	0.479, 0.634	−0.169, 0.276	7.13 ±0.38	7.28 ±0.48	−1.301, 0.198	−0.372,0.079	6.67 ±0.37	7.16 ±0.47	−4.411, 0.000	−0.712, −0.268
Rational Ability	2.89 ±0.26	3.01 ±0.25	−1.798, 0.077	−0.254, 0.014	3.40 ±0.29	2.89 ±0.28	6.856, 0.000	0.363, 0.663	3.87 ±0.34	3.03 ±0.35	9.308, 0.000	0.662, 1.025
Rational Engagement	3.05 ±0.28	3.08 ±0.25	−0.432, 0.667	−0.169, 0.109	3.43 ±0.28	3.11 ±0.27	4.450, 0.000	0.178, 0.469	3.70 ±0.30	3.15 ±0.26	7.490, 0.000	0.401, 0.693
Experiential Ability	3.88 ±0.30	3.78 ±0.32	−1.553, 0.126	−0.343, 0.043	3.68 ±0.27	3.81 ±0.30	−1.780, 0.080	−0.283,0.017	3.37 ±0.34	3.66 ±0.30	−3.398, 0.001	−0.456, −0.118
Experiential Engagement	3.66 ±0.28	3.72 ±0.26	−0.746, 0.459	−0.0197, 0.090	3.45 ±0.24	3.47 ±0.29	−0.188, 0.851	−0.155, 0.128	3.30 ±0.24	3.50 ±0.30	−2.869, 0.006	−0.345, −0.061
Perceptual-motor processing speed												
Finding As task ^d^	1548.47 ±429.81	1505.90 ±384.77	0.404, 0.688	−168.260, 253.394	1375.00 ±251.88	1445.03 ±336.34	−0.913, 0.365	−223.603, 83.536	1206.47 ±282.06	1417.70 ±284.05	−2.890, 0.005	−357.530, −64.937
Copying ^e^	1276.87 ±408.56	1517.80 ±429.44	−2.226, 0.030	−457.559, −24.307	1132.97 ±292.41	1447.30 ±280.88	−4.246, 0.00	−462.516, −166.150	987.63 ±270.10	1406.07 ±290.16	−5.781, 0.000	−563.310, −273.556
Number ^e^ comparison	590.03 ±207.11	618.93 ±247.10	−0.491, 0.625	−146.734, 88.934	493.63 ±142.89	573.03 ±219.99	−1.658, 0.10	−175.271, 16.471	405.93 ±95.25	554.43 ±183.71	−3.930, 0.000	−224.130, −72.870

Note: Values are expressed as means ± standard deviations. ^a^: Examined by independent *t*-test between groups; ^b^: CFQ, Cognitive Failure Questionnaire; ^c^: REI, Rationale-Experiential Inventory; ^d^: Unit of time, s = second; ^e^: Unit of time, cs = centisecond; ICB-VIP: Integrated Cognitive Based Violence Intervention Program; ICB-VIP is abbreviated as ICB in table; TAU: treatment as usual.

**Table 4:** Pre-post-intervention and follow-up data for social cognition and emotion regulation outcomes.

ICB-VIP (ICB) vs. TAU	Baseline (Time 1)				Post-Intervention (Time 2)				Follow-Up (Time 3)			
	ICB	TAU	*t*, *p* ^a^	95% CIs	ICB	TAU	*t*, *p*^a^	95% CIs	ICB	TAU	*t*, *p*^a^	95% CIs
Alexithymia												
DIF	24.63 ±3.13	25.97 ±3.23	−1.622, 0.110	−2.979, 0.312	21.67 ±3.54	24.00 ±3.94	−2.408, 0.019	−4.273, −0.394	18.99 ±3.79	23.36 ±4.31	−4.171, 0.000	−6.477, −2.276
DDF	19.50 ±3.49	18.53 ±3.13	1.128, 0.264	−0.749, 2.682	15.22 ±2.60	16.79 ±2.90	−2.202, 0.032	−2.991, −0.142	14.27 ±2.98	15.06 ±2.44	−1.110, 0.272	−2.189, 0.627
EOT	26.00 ±3.28	24.80 ±3.22	1.430, 0.158	−0.480, 2.880	23.33 ±3.72	25.00 ±3.15	−1.871, 0.066	−3.450, 0.117	20.85 ±3.25	23.03 ±4.03	−2.307,0.025	−4.082, −0.0290
Total	70.13 ±6.95	69.30 ±5.10	0.529, 0.599	−2.318, 3.984	60.22 ±7.28	65.79 ±4.95	−3.462, 0.001	−8.785, −2.348	54.11 ±6.13	61.45 ±7.17	−4.261, 0.000	−10.792, −3.893
Emotion regulation												
Reappraisal	19.87 ±2.75	20.83 ±2.73	−1.366, 0.177	−2.383, 0.450	24.17 ±2.45	23.20 ±2.48	1.518, 0.135	−0.308, 2.242	26.87 ±1.88	22.07 ±3.30	6.914, 0.000	3.410, 6.190
Suppression	14.13 ±2.43	13.10 ±2.68	1.563, 0.123	−0.290, 2.357	10.50 ±3.82	12.83 ±2.64	−2.751, 0.008	−4.031, −0.636	9.67 ±3.70	13.20 ±2.20	−4.487, 0.000	−5.110, −1.957

Note: Values are expressed as means ± standard deviations. ^a^: Examined by independent *t*-test between groups; ICB-VIP (ICB): Integrated Cognitive Based Violence Intervention Program; ICB-VIP is abbreviated as ICB in table; TAU: treatment as usual; ERQ: Emotional Regulation Questionnaire; DIF: difficulty in identifying feelings; DDF: difficulty in describing feelings; EOT: externally orientated thinking.

**Table 5:** Repeated-measures effects of the Integrated Cognitive Based Violence Intervention Program on study outcomes.

	Group (Inter-Group) Effect		Time (within) Effect		Time and Group Interaction	
	F	*p*	F	*p*	F	*p*
Aggression	9.592	0.003	20.925	0.000	12.311	0.001
Violence attribution	0.883	0.351	20.492	0.000	12.500	0.001
Clinical symptoms	7.265	0.009	263.898	0.000	27.393	0.000
Cognitive failure	8.236	0.006	85.290	0.000	31.722	0.000
Reasoning and decision-making						
Rational	60.187	0.000	171.245	0.000	137.278	0.000
Experiential	3.729	0.058	128.141	0.000	25.838	0.000
Perceptual-motor processing speed						
Finding As task	1.395	0.242	14.906	0.000	5.188	0.026
Copying	18.301	0.000	19.937	0.000	3.907	0.053
Number comparison	4.337	0.042	22.170	0.000	5.131	0.027
Alexithymia	10.417	0.002	121.030	0.000	14.195	0.000
Emotion regulation						
Reappraisal	11.993	0.001	85.303	0.000	41.847	0.000
Suppression	5.936	0.018	37.688	0.000	41.219	0.000

## Data Availability

Data are available on request from the authors.
